# Severe Acute Respiratory Syndrome–associated Coronavirus in Lung Tissue

**DOI:** 10.3201/eid1001.030404

**Published:** 2004-01

**Authors:** Tony Mazzulli, Gabriella A. Farcas, Susan M. Poutanen, Barbara M. Willey, Donald E. Low, Jagdish Butany, Sylvia L. Asa, Kevin C. Kain

**Affiliations:** * Mount Sinai Hospital, Toronto, Ontario, Canada; † Toronto Medical Laboratories, Toronto Ontario, Canada; ‡ University of Toronto, Toronto, Ontario, Canada; § Toronto General Hospital, Toronto, Ontario, Canada; ¶ University Health Network, Toronto, Ontario, Canada

**Keywords:** SARS, coronavirus, lung tissue, Canada, RT-PCR

## Abstract

Efforts to contain severe acute respiratory syndrome (SARS) have been limited by the lack of a standardized, sensitive, and specific test for SARS-associated coronavirus (CoV). We used a standardized reverse transcription-polymerase chain reaction assay to detect SARS-CoV in lung samples obtained from well-characterized patients who died of SARS and from those who died of other reasons. SARS-CoV was detected in all 22 postmortem lung tissues (to 10^9^ viral copies/g) from 11 patients with probable SARS but was not detected in any of the 23 lung control samples (sample analysis was blinded). The sensitivity and specificity (95% confidence interval) were 100% (84.6% to 100%) and 100% (85.1% to 100%), respectively. Viral loads were significantly associated with a shorter course of illness but not with the use of ribavirin or steroids. CoV was consistently identified in the lungs of all patients who died of SARS but not in control patients, supporting a primary role for CoV in deaths.

From its origins in November 2002 in Guangdong Province, China, severe acute respiratory syndrome (SARS) has become an emerging infectious disease that has spread to areas throughout the world, including Hong Kong, Vietnam, Singapore, Taiwan, and Canada (*1*). Although controversy remains over the etiology of SARS, the World Health Organization has declared a newly described virus known as the SARS-associated coronavirus (SARS-CoV) as the cause (*2*). This announcement has led to a rapid proliferation of different in-house laboratory tests aimed at detecting either SARS-CoV–specific antibodies or SARS-CoV nucleic acid in clinical specimens. The Centers for Disease Control and Prevention definition for a confirmed case of SARS includes the results of these laboratory tests (*3*). However, because different assays are being used, comparing results from different centers has been difficult. In addition, the inability of these nonstandardized tests to detect SARS-CoV in all cases has led to speculation that other agents may be associated with SARS. Some researchers have suggested that illnesses that progress to respiratory failure and death may not be caused by uncontrolled viral replication but rather are the result of an immunopathologic process (*4*). In a recent report of six fatal cases of SARS, SARS-CoV was detected by reverse transcriptase-polymerase chain reaction (RT-PCR) in postmortem lung tissue in only four patients (*5*).

The purpose of this study was to use a standardized, commercially available, RT-PCR assay to test for the presence of SARS-CoV RNA. Lung tissue obtained at autopsy from well-characterized patients with SARS who died during the outbreak in Canada were compared to lung samples obtained at autopsy from patients without SARS who died during the outbreak and lung samples from patients who died before the outbreak.

## Methods

### Patients

All patients who met the current World Health Organization case definition of probable SARS and who underwent a postmortem examination in Canada during the March–April 2003 outbreak were included in this study. Clinical details were extracted retrospectively from hospital records. Clinical descriptions of some of these cases have been published separately (*6**,**7*). As of May 14, 2003, a total of 24 patients died of SARS in Canada; all died in Toronto. Of the 24 patients, autopsies were performed on 11 patients. Results of ante- and postmortem examination for routine bacterial and viral respiratory pathogens from these 11 patients, as described elsewhere, were negative (*6*).

### Lung Tissue Samples

A total of 22 discrete postmortem lung samples collected from these 11 patients were included in this analysis. An additional 13 postmortem lung samples from 7 patients who died during the SARS outbreak but whose deaths were attributed to other causes were also included. The attributed cause of death in these patients was as follows: a 46-year-old woman died of invasive group A streptococcal infection; a 93-year-old man died of congestive heart failure; a 37-year-old man died of sudden death cardiovascular disease; a 74-year-old man died of amiodarone pulmonary toxicity; a 78-year-old woman died of dementia and aspiration pneumonia; a 47-year-old woman died of diabetes and congestive heart failure; and an 81-year-old man died of bladder cancer and aspiration pneumonia. In addition, 10 lung samples collected in 1998 from 10 patients (4 women and 6 men; age range 54–75 years) with lung cancer were also included as negative controls. All samples collected at the time of autopsy were snap frozen in a mixture of absolute ethanol and dry ice and subsequently stored at –70°C until tested. The samples were coded and then processed, subjected to RT-PCR analysis, and interpreted before the identity of the samples was divulged. This study was approved by the research ethics boards at Mount Sinai Hospital and the University Health Network.

### RT-PCR

Lung tissue samples were thawed and immediately homogenized in lysis buffer (QIAGEN, Mississauga, Canada) with disposable tissue grinders (Kendall Precision, Mansfield, MA). The homogenate was passed through QIAshredder columns (QIAGEN) before RNA isolation by using the RNeasy Mini Kit (QIAGEN). The sample was eluted in 30 μL of RNAse free water. The RT-PCR was carried out by using the RealArt HPA-Coronavirus LightCycler RT Reagents Assay (Artus GmbH, Hamburg, Germany) with a LightCycler real-time platform (Roche Diagnostics, Laval, Canada). The HPA-Coronavirus Master Mix contains reagents and enzymes for the specific amplification of an 80-bp region of the SARS-CoV polymerase gene from 5 μL of RNA with the primer pairs published by the Bernhard-Nocht Institute (Hamburg, Germany) as posted on the World Health Organization Web site (available from: URL: http://www.who.int/csr/sars/primers/en/).

Viral load was calculated from a standard curve based on four external positive controls (quantification standards) included in the RealArt HPA-Coronavirus LightCycler RT Reagents Assay kit (Figure A and B). The standards were treated as previously purified samples, and the same 5-μL volume was added per capillary. A standard preparation of SARS-CoV isolated from cell culture supernatants of VeroE6 cells was used as a calibrator in each run. In addition, the kit contains a second heterologous amplification system (i.e., an internal control) to identify either PCR inhibition exclusively, if added to the extracted RNA, or RNA isolation quality as well as PCR inhibition, if added during the RNA isolation procedure (Figure C). Although the assay insert states that the primers and probes used in the assay were checked for possible similarity to other pathogens by means of sequence comparison, 25 randomly chosen amplicons from our sample pool were independently sequenced to confirm SARS-CoV–specific amplification and detection. Univariate analysis comparing potential predictors of viral load (e.g., duration of illness, the use of ribavirin, the use of steroids) was completed by using Fisher exact test. Two-sided p values <0.05 were considered significant.

**Figure:**
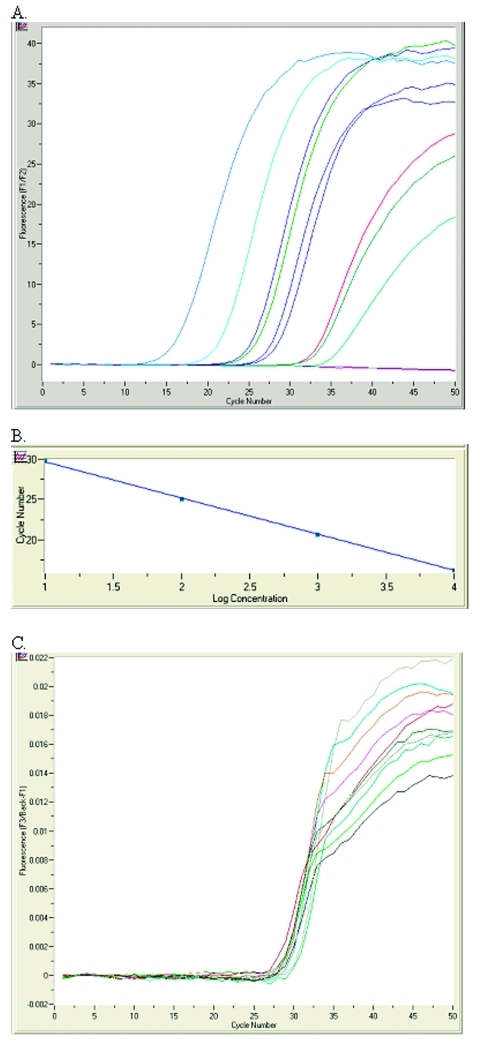
RealArt HPA-Coronavirus LightCycler (RealArt HPA Coronavirus RT-PCR) reverse transcription-polymerase chain reaction (PCR) Assay results. PCR results from 5 μL RNA are displayed in channel F1/F2 of the LightCycler instrument (A). Four quantification standards are included in the assay to generate a standard curve (B). An internal control, added at the RNA isolation stage, is used to monitor both the quality of the RNA isolation as well as possible PCR inhibition (C).

## Results

The clinical description and RT-PCR results for the 11 patients with probable SARS from whom postmortem lung tissue samples were examined are summarized in Table 1. The mean age of the 11 patients was 70 years (range 43–99). Six of the 11 patients were men. All but 1 of the 11 patients had underlying coexisting conditions, the most common of which was diabetes mellitus in 6 patients. The mean duration of illness was 20 days (range 8–32). Seven patients had been intubated and mechanically ventilated before death. Three patients had requested not to be intubated (information on ventilation was not available for one patient). Ten of the 11 patients were treated with ribavirin; 6 of the 11 patients were treated with steroids.

**Table 1 T1:** Clinical description and SARS-CoV RT-PCR results for 11 patients who died with probable SARS^a^

Sex/age	Coexisting conditions	Illness and treatment duration (days)				Postmortem lung tissue description	RealArt HPA Coronavirus RT-PCR^b^	
		Illness	Ventilation	Ribavirin	Steroids		Results	Copies of CoV/ gram tissue
M/43	Type II DM, HTN	15	4	0	0	RUL	Positive	1.5 x 10^8^
						RML (#1)	Positive	5.4 x 10^7^
						RML (#2)	Positive	2.8 x 10^7^
						RML (#3)	Positive	7.4 x 10^6^
						RML (#4)	Positive	6.4 x 10^8^
M/76	Type II DM, CAD, HTN	11	4	6 (started on day 6 of illness)	0	Lung	Positive	3.8 x 10^9^
F/78	Type II DM, CAD, hypercholesterolemia, chronic obstructive pulmonary disease	8	5	>5 (started on day 4 of illness)	0	RT lung	Positive	1.0 x 10^9^
						LUL	Positive	9.4 x 10^7^
M/62	Rectal cancer; HTN, hypercholesterolemia	8	N/A	>5 (started on day 4 of illness)	0	LT lung	Positive	5.3 x 10^7^
F/73	HTN, hypercholesterolemia	28	DNI	14 (started on day 5 of illness)	12 (stated on day 14 of illness)	LT lung	Positive	3.0x 10^4^
						RT lung	Positive	3.6 x10^5^
F/99	Osteoarthritis	26	DNI	13 (started on day 1 of illness)	0	Lung	Positive	5.0 x 10^4^
M/63	Hypercholesterolemia, cerebral vascular disease	20	12	16 (started on day 4 of illness)	16(started on day 6 of illness)	RUL lung	Positive	3.2 x 10^6^
						LLL	Positive	2.5 x10^7^
F/78	Type II DM, HTN, hypercholesterolemia	24	18	10 (started on day 3 of illness)	18 (started on day 5 of illness)	LT lung	Positive	4.1 x 10^5^
						RUL	Positive	4.9x10^5^
M/44		29	18	18 (started on day 8 of illness)	17 (started on day 12 of illness)	RT lung	Positive	7.6 x 10^4^
						LT lung	Positive	4.1 x 10^4^
M/77	Type II DM, HTN, hypercholesterolemia	>18	>1	>1 (started on day 10 of illness)	>7 (started on day 10 of illness)	LLL	Positive	5.6 x 10^5^
						LUL	Positive	5.7 x 10^5^
F/79	Type II DM, HTN, hypercholesterolemia	32	DNI	11 (started on day 2 of illness)	>4 (started on day 12 of illness)	LT lung	Positive	2.7 x 10^4^
						Lung	Positive	2.1 x 10^5^

^a^SARS, severe acute respiratory syndrome; CoV, coronavirus; RT-PCR, reverse transcription polymerase chain reaction; F, female; M, male; DM, diabetes mellitus; HTN, hypertension; RUL, right upper lobe; RML, right middle lobe; CAD, coronary artery disease; RT, right; LT, left; LUL, left upper lobe; LLL, left lower lobe; N/A, not available; DNI, “Do not intubate” order written. ^b^RealArt HPA Coronavirus RT-PCR (Artus GmbH, Hamburg, Germany).

SARS-CoV was detected in all 22 postmortem lung tissue samples collected from all 11 patients who died with a diagnosis of probable SARS. All 13 postmortem lung samples from the seven non-SARS fatalities that occurred during the SARS outbreak were negative for SARS-CoV, as were all 10 lung-tissue samples collected from patients with lung cancer 5 years before the outbreak (Table 2)[Table T1]. The corresponding sensitivity and specificity of the RealArt HPA-Coronavirus LightCycler RT Reagent assay are both 100% (95% confidence interval [CI] for sensitivity 84.6% to 100%; 95% CI for specificity 85.1% to 100%) for the detection of SARS-CoV.

**Table 2:** Univariate analysis of predictors of high viral loads in postmortem lung tissue

Predictor	Viral load >10^6^ copies/g lung tissue	Viral load <10^6^ copies/g lung tissue	Fisher exact test
Short duration of illness (<21 d)	5/5	0/6	p=0.002
Use of ribavirin	4/5	6/6	p=0.45
Use of steroids	1/5	5/6	p=0.08

The SARS-CoV viral load in postmortem lung tissue ranged from 2.7 x 10^4^ copies/g tissue to 3.8 x 10^9^ copies/g tissue. Higher viral loads (>10^6^ copies/g tissue) were associated with patients who had a shorter duration of illness (<21 days) (p=0.002, Fisher exact test). The use of ribavirin or steroids was not significantly associated with viral load levels (Table 2)[Table T1].

Twenty-five randomly selected amplicons from the sample pool were sequenced to assess specificity and possible cross-reactivity to other pathogens. A BLAST (available from: URL: http://www.ncbi.nlm.nih.gov/BLAST/) search performed against the SARS-CoV genomes in GenBank, European Molecular Biology Laboratory, DNA Data Bank of Japan, and Protein Data Bank on the National Center for Biotechnology Information Web site (available from: URL: http://www.ncbi.nlm.nih.gov/), indicated that all amplicon samples contained SARS-CoV polymerase gene sequence.

## Discussion

By using a standardized RT-PCR assay, SARS-CoV has been unequivocally identified in the lung tissue of all patients who died with probable SARS but not in any of the controls. These observations support a primary role for this virus in patients with SARS who have fatal outcomes and provide additional, strong evidence to fulfill Koch’s postulates regarding SARS-CoV as the cause of SARS (*8*). SARS-CoV was found in different lung samples from the same patient, suggesting that the virus is widely disseminated throughout the lung at the time of death. Previous studies suggested that progression of disease to respiratory failure may be primarily mediated by host immune response rather than viral replication (*4*). Although viral RNA in lung tissue does not necessarily indicate replicating virus, virus in multiple lung lobes, often in high copy number, at the time of death suggests that SARS-CoV may also be contributing to disease progression. The fact that higher viral loads were significantly associated with patients with a shorter duration from onset of illness to death supports the role of viral replication as a contributor to death. Ten of the 11 patients had received therapy with ribavirin, and 6 patients were treated with steroids. The failure to eradicate SARS-CoV despite ribavirin therapy and the lack of association between the use of ribavirin and SARS-CoV viral load are consistent with in vitro data showing that ribavirin has no activity against this agent (*9*).

Global efforts to contain SARS have been severely impeded by the lack of a standardized, sensitive, and specific diagnostic test for SARS-CoV. Different diagnostic strategies, including culture, serologic assays, and molecular detection methods, have been described, but each of these tests has limitations. In-house RT-PCR assays have been associated with sensitivities as low as 50% in patients with SARS (*10*), which raises uncertainty about the role of CoV versus co-pathogens in mediating severe or fatal SARS. By contrast, the sensitivity and specificity of the RealArt HPA-Coronavirus RT-PCR assay for detecting CoV in lung tissue samples appear to be excellent. In addition, with the real-time LightCycler system, the assay generates quantitative results within 1 hour, which is much shorter than traditional PCR reactions.

The type of specimen tested, the timing of sample collection, (i.e., acute versus convalescent phase) the method of specimen collection, as well as the method of sample preservation may have substantial impact on the results obtained from a diagnostic test. The lower sensitivity of SARS-CoV detection reported by Peiris et al. (*10*) may be a consequence of these confounding factors. Our study design of examining lung biopsies from clearly defined patient populations overcame confounding issues, such as sampling technique, nonspecific case definitions, and possible undocumented exposure to SARS. Given the predominance of respiratory symptoms in patients with SARS, lung samples have perhaps the highest viral titers of all specimen types; yet in nonfatal cases, obtaining routine lung biopsies is not practical. Other respiratory tract specimens may be satisfactory substitutes for biopsies, but further studies examining the prevalence of SARS-CoV in these other specimen types and in a larger population are needed. With the use of standardized commercially available assays, comparison of results from different centers may be facilitated.
